# Remote Limb Ischemic Postconditioning Protects against Ischemic Stroke via Modulating Microglia/Macrophage Polarization in Mice

**DOI:** 10.1155/2021/6688053

**Published:** 2021-02-19

**Authors:** Dong Han, Jue Wang, Lulu Wen, Miao Sun, Hang Liu, Yan Gao

**Affiliations:** Department of Neurology, Shengjing Hospital of China Medical University, 36 Sanhao Street, Heping District, Shenyang 110004, China

## Abstract

**Aim:**

The protection against ischemia/reperfusion injury mediated by remote limb ischemic postconditioning (RIPC) shows great clinical value in ischemic stroke therapy, but the particular mechanism of RIPC remains unclear.

**Methods:**

We carried out middle cerebral artery occlusion/reperfusion (MCAO/R) surgery on C57BL/6 male mice. RIPC was generated by 10-minute occlusion followed by the same period of reperfusion of the bilateral hind limb femoral artery and repeated for 3 cycles. Infarct size and neurological score were performed to assess stroke outcomes. Ly6C^hi^ monocytes were quantified in the blood and brain by flow cytometry. Real-time PCR, ELISA, and immunofluorescence were utilized to detect phenotype of proinflammatory M1 and anti-inflammatory M2 microglia/macrophage. Nuclear factor *κ*B (NF-*κ*B) and peroxisome proliferator-activated receptor *γ* (PPAR*γ*) levels were detected using Western blot.

**Results:**

At 24 and 72 h after MCAO, RIPC drastically attenuated infarct size and ameliorated the neurological deficits of mice and facilitated transmigration of Ly6C^hi^ monocytes to the brain postischemia reperfusion. Furthermore, RIPC contributed to increased M2 and reduced M1 microglia/macrophage through inhibiting NF-*κ*B and promoting PPAR*γ* activation.

**Conclusion:**

Our results reveal pharmacological effect of RIPC in promoting microglia/macrophage transferring from M1 to M2 phenotype after MCAO/R in mice, which provides theoretical support for the therapeutic effect of RIPC in ischemic stroke.

## 1. Introduction

Despite significant advances in medical and surgical intervention, ischemic stroke is still a prominent source of death and long-term disability globally. For patients with acute ischemic stroke, besides timely and effective reperfusion, adjunct therapies are also required to ameliorate the clinical outcomes and decrease infarct sizes. Remote limb ischemic postconditioning (RIPC) is an effective protection strategy that performs ischemic condition on a remote organ (limbs) after cerebral ischemia to protect the brain from ischemia/reperfusion injury in models of experimental stroke. Despite the fact that neuroprotection of RIPC has been validated across several studies [1, 2], the underlying mechanisms of its effects have not been clarified.

More and more evidences indicate that microglia/macrophages play a critical function in modulating immune and inflammatory responses [3]. After cerebral ischemia, a large number of microglia are rapidly activated and polarized into two different phenotypes, namely, “classical activation” (M1 phenotype) and “alternative activation” (M2 phenotype) [4]. M1 microglia/macrophages secrete proinflammatory cytokines and neurotoxic mediators, such as tumor necrosis factor- (TNF-) *α*, interleukin- (IL-) 1*β*, and IL-6. The significant production of these inflammatory cytokines participates in the pathogenesis of stroke and aggravate neuronal injury [5]. In M1 microglia/macrophage, nuclear factor *κ*B (NF-*κ*B) activation may facilitate the expression of proinflammatory cytokines, leading to neurotoxic outcomes [6]. In comparison to the M1 phenotype, M2 microglia/macrophages exert anti-inflammatory effects, encourage wound healing, and ameliorate tissue repair [5]. Multiple anti-inflammatory cytokines, including IL-4, IL-10, transforming growth factor-*β* (TGF-*β*), and arginase-1 (Arg-1), have been shown to improve inflammatory responses and increase gene expression that plays a role in tissue recovery [7–9]. Thus, swapping microglia/macrophage from an M1 to an M2 phenotype may represent an efficacious treatment strategy for ischemic stroke therapy.

The peroxisome proliferator-activated receptors (PPARs) belong to ligand-induced nuclear receptors. According to the structure, PPARs are divided into three subtypes, including PPAR*α*, PPAR*β*/*δ*, and PPAR*γ*. PPARs bind to ligands, then form heterodimer complex with retinol X receptors. After that, the complex binds to the PPAR response element, which is upstream of the target gene promoter, and finally modulates gene transcription [10]. PPAR*γ* was first reported to have a significant function in fat formation and glycolipid metabolism [11]. Recently, several researches have shown that activating PPAR*γ* signaling leads to a reduction in inflammation which is related to regulation of the M1/M2 phenotype in Alzheimer's disease and experimental stroke [9, 12].

In this study, we explored the therapeutic efficacy of RIPC by identifying the inflammatory cytokines and M1/M2 polarization markers that are present in the cortex of middle cerebral artery occlusion/reperfusion (MCAO/R) mice and also investigated whether the neuroprotective effect is associated with the NF-*κ*B and PPAR*γ* pathway.

## 2. Materials and Methods

### 2.1. Animals and Treatment

A total of one hundred and thirty heathy male C57BL/6 mice (20-25 g) were purchased from the Beijing HFK Bioscience Corporation, China. Mice were maintained 5 per cage in a temperature-controlled room (22 ± 2°C), at a 12 h light-dark cycle and a relative humidity of 60 ± 10%. The mice were randomly allocated to 3 groups: sham, MCAO, and MCAO+RIPC group. Then, all mice were euthanized at 24 h or 72 h post-MCAO, respectively. In order to assess neurological deficit, infarct size, and ELISA analyses,*n* = 6 for each group were utilized. For real-time PCR, *n* = 4 per group. For immunofluorescence, flow cytometry analysis, and Western blot, *n* = 3 for each group. Twenty-two mice died from intracranial hemorrhage, and the data from these mice were excluded in the analysis. The protocols for all animal experiments were approved by the Institutional Animal Care and Use Committee of China Medical University (Shenyang, China).

### 2.2. Focal Cerebral Ischemia

Ischemic stroke was performed as previously described utilizing the mouse model of MCAO with some modifications [13]. Briefly, the right common carotid artery and external and internal carotid artery were exposed, and a silicon-coated monofilament nylon suture (diameter of 0.22 ± 0.01 mm, Beijing Cinontech Co. Ltd., China) was inserted into the internal carotid artery to occlude the origin of MCA, under isoflurane anesthesia. The filament was removed after 1 h to initiate reperfusion. During the surgery, core body temperatures were continuously maintained at 36.8–37.2°C. The sham group was performed the same surgery with the exception of the MCA occlusion.

### 2.3. Remote Limb Ischemic Postconditioning

RIPC was performed as previously described [14]. In brief, RIPC was accomplished using three cycles of bilateral hind limb ischemia (10 min/cycle, 50 min total). For every cycle, the proximal region of the hind limbs was secured using a tourniquet for 10 min, followed by reperfusing for 10 min with the tourniquet released. RIPC was conducted immediately after MCAO/R surgery. The sham and MCAO groups contain all surgical techniques, excluding the cycles of bilateral hind limb ischemia and reperfusion.

### 2.4. Neurological Scoring

The mice were given scores according to their grades of neurological deficit at 24 h or 72 h postreperfusion. The neurological scores were evaluated using the method of Bederson et al. [15]. While being held suspended by the tail, mice that lengthened both forelimbs towards the floor without additional neurological deficits were rated 0. Mice that fail to extend consistent forelimb fully with the injured hemisphere were assigned grade 1. When being placed on a smooth surface, mice that had decreased resistance to lateral push on the shoulder towards the paretic side were assigned grade 2. Mice that circled to the contralateral side were rated 3. Mice that flaccid paralysis without spontaneous movements were allocated a grade of 4.

### 2.5. Measurement of Infarct Size

Mice were euthanized and their entire brains were rapidly extracted at 24 h or 72 h after surgery. Each brain was dissected and coronally sectioned 5 slices at 2 mm intervals. Place brain slices in 1% 2, 3, 5-triphenyltetrazolium chloride (TTC, Sangon Biotech Co., Ltd., China) solution and incubate for 10 min at 37°C in the dark [16]. The infarct size on both sides of each section was evaluated utilizing the ImageJ software (version, 1.50; National Institutes of Health, USA). The infarct size was represented as an average percentage from the 5 slices according to this equation:

(1) $$\begin{array}{c} \text{Infarct size }\left(\%\right)=\left(\text{contralateral area}-\text{ipsilateral noninfarct area}\right)/\text{contralateral area}\times 100\%. \end{array}$$

### 2.6. ELISA

Tissue samples for ELISA assay were gathered through the ischemic hemisphere after execution. Samples were gathered after centrifuging the tissue homogenate for 10 min at 5000 × g. The levels of inducible nitric oxide synthase (iNOS), CD86, Arg-1, and CD206 were measured by using corresponding ELISA kits (Shanghai Enzyme-linked Biotechnology Co., Ltd., China).

### 2.7. Real-Time PCR

The levels of iNOS, TNF-*α*, Arg-1, and TGF-*β* mRNA in each group were determined by real-time PCR as previously described [17]. Total RNA in ischemic brain tissue was removed utilizing Trizol reagent (Vazyme Biotech Co., Ltd., China). As per manufacturer's guidelines, RNA was reverse-transcribed into cDNA utilizing HiScript II Q RT SuperMix kit; real-time PCR was conducted through ChamQ SYBR qPCR Master Mix kit in quantitative PCR Mastercycler (Eppendorf, Germany). The two kits mentioned above were purchased from Vazyme Biotech Co., Ltd. (China). The primer sequences (Sangon Biotech Co., Ltd., China) were as follows:

*iNOS*: F: AATGGCAACATCAGGTGGCCATCACT, R: GCTGTGTGTCACAGAAGTCTCGAACTC;

*TNF-α*: F: GCACCACCATCAAGGACTCA, R: TCGAGGCTCCAGTGAATTCG;

*Arg-1*: F: GAACACGGCAGTGGCTTTAAC, R: TGCTTAGTTCTGTCTGCTTTGC;

*TGF-β*: F: TGGCTGAACCAAGGAGACG, R: GCAGTGAGCGCTGAATCGA;

*β-Actin*: F: CATCCGTAAAGACCTTTGCCAAC, R: ATGGTGCCACCGATCCACA.

### 2.8. Immunofluorescence

At 24 h or 72 h after surgery, animals were deeply anaesthetized and perfused with saline, followed by 4% paraformaldehyde. The brains were gathered and dehydrated in 30% sucrose solution overnight. After that, the brains were placed in OCT and cut into 20 *μ*m frozen segments. The slices were placed in blocking buffer in 8% goat serum for 2 h at room temperature and then treated with a primary antibody: mouse anti-Iba1 (1 : 100, Santa Cruz Biotechnology, USA), rabbit anti-CD16 (1 : 100, Abcam, USA), and rabbit anti-CD206 (1 : 100, Abcam, USA) at 4°C overnight. Membranes were washed with PBST three times (0.1% tween 20 in PBS); the tissue sections were treated with FITC- or Cy3-conjugated secondary antibody (1 : 100, BIOSS, China) and at room temperature for 2 h. Finally, nuclei were costained with DAPI (Beyotime Institute of Biotechnology, China) for 30 min. Images were obtained through a laser scanning confocal microscope (LSM700, Carl Zeiss, Germany) and assessed using ImageJ software. The stained cells were counted in the ipsilateral cortex penumbra under 400x magnification.

### 2.9. Flow Cytometry Analysis

The brain was removed, and the ischemic hemisphere was collected for flow cytometry. Monocyte classification by phenotypic analysis through expression of cell surface antigens was conducted using flow cytometry [18]. All antibodies were bought through BioLegend (USA), including fluorescein isothiocyanate (FITC) anti-mice Ly6C (128005), phycoerythrin (PE) anti-mice CD11b (101207), and PE-Cy5 conjugate (PE/Cy5) anti-mice CD45 (103109). Cells were analyzed on a Miltenyi Biotec MACSQuant flow cytometer (Germany). Cells were gated on the scatter plots of forward scatter (FSC-H) and side scatter (SSC-H), with the exclusion of debris and cell aggregates. The total number of leukocytes was gated as CD45^hi^ populations. Monocytes were identified as CD45^hi^ CD11b^+^ Ly6C^+^ populations and further separated as Ly6C^hi^ or Ly6C^lo^.

### 2.10. Western Blot

To identify NF-*κ*B and PPAR*γ* protein levels in ischemic brain, protein from the nucleus and cytoplasm was isolated utilizing the Nuclear and Cytoplasmic Extract Kit (Solarbio, China). Next, concentration of total protein was evaluated using BCA assay (Applygen Technologies Inc., China). Equivalent levels of protein were separated through SDS-PAGE and transferred onto polyvinylidene fluoride (PVDF) membranes. After treatment with blocking buffer (5% skim milk in TBST) for 1.5 h at room temperature, the membranes were probed with primary antibodies targeting PPAR*γ* (1 : 1000, Cell Signaling Technology, USA), NF-*κ*B (1 : 1000, Proteintech Group, USA), *β*-actin (1 : 3000, Abways Technology, China), and H3 (1 : 1000, Abways Technology, China) overnight at 4°C. After being washed three times with TBST, the membranes were probed with horseradish peroxidase- (HRP-) conjugated secondary antibodies (1 : 5000, Abways Technology, China) for 2 h. Relative band densities were subsequently observed using ECL kit (Applygen Technologies Inc., China) on a chemiluminescence imaging system (Bio-Rad, USA). Protein levels were measured with ImageJ, and the data was normalized to anti-*β*-actin or anti-H3.

### 2.11. Statistical Analysis

All data were evaluated or quantified in a completely blind manner. IBM SPSS Statistics v19.0 software was used to perform statistical analyses. Statistical variation between groups was determined utilizing one-way analysis of variance (ANOVA). The neurological scores and real-time PCR were compared with other groups using the Mann-Whitney *U* test. The comparative variation was significant at *P* < 0.05. Statistical figures were presented using ImageJ, Photoshop CS6, and GraphPad Prism version 5.0. The data was presented as mean ± SD.

## 3. Result

### 3.1. RIPC Attenuated Ischemic Outcome Both at 24 H and 72 H in Mice after MCAO

**Figure 1:**
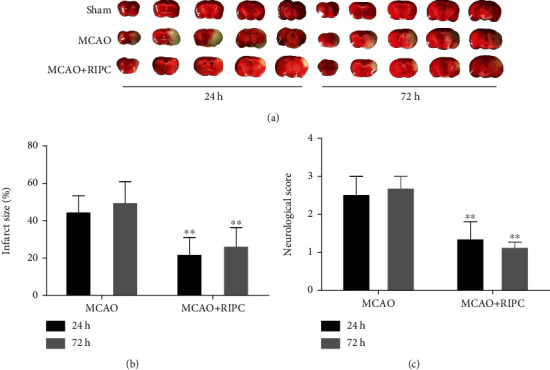
RIPC reduced infarct size and ameliorated neurological behavior at 24 h and 72 h after MCAO: (a) representative images of TTC-stained brain sections; (b) brain infarct size; (c) neurological function scores. Data presented as mean ± SD (*n* = 6). ^∗^*P* < 0.05 and ^∗∗^*P* < 0.01*vs.* the MCAO group.

The infarct size and neurological defect were detected in different groups at 24 h and 72 h postreperfusion. As depicted in Figure 1(b), the infarct size of the MCAO+RIPC group significantly decreased in comparison to the MCAO group at 24 h and 72 h (*P* < 0.01). The consequence of RIPC on neurological dysfunction stimulated by MCAO was assessed. The scores of the sham group were zero, which showed no neurological deficits. The scores increased in the MCAO group, while those of the MCAO+RIPC group decreased significantly in comparison with the MCAO group at 24 h and 72 h (*P* < 0.01, Figure 1(c)). These results suggested that RIPC reduced infarct size and improved neurological deficit score at 24 h and 72 h after stroke in mice.

### 3.2. RIPC Upregulated Ly6C^hi^ Monocyte Infiltration into Ischemic Brain in Mice after MCAO

**Figure 2:**
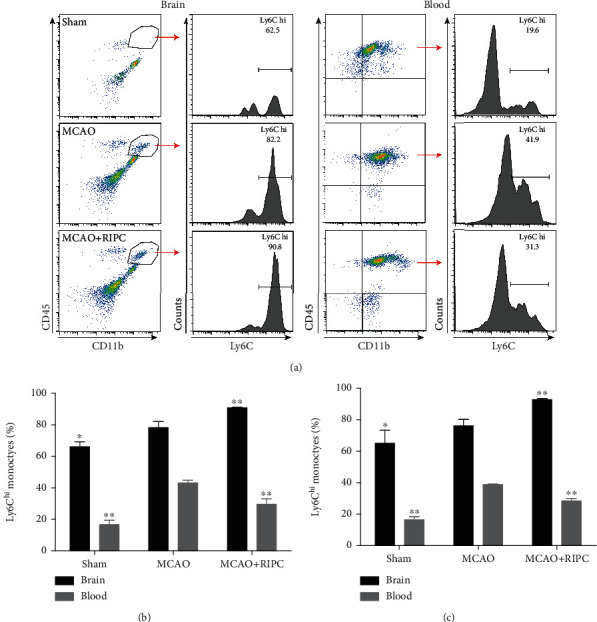
RIPC accelerated the infiltration of Ly6C^hi^ monocytes into ischemic brain at 24 h and 72 h after MCAO. (a) Representative gating strategy for flow cytometric analysis of Ly6C^hi^ monocytes in the brain and blood at 24 h. Quantification of the percentage of Ly6C^hi^ monocytes in the brain and blood at 24 h (b) and 72 h (c). Data presented as mean ± SD (*n* = 3). ^∗^*P* < 0.05 and ^∗∗^*P* < 0.01*vs.* the MCAO group.

We use CD45 and CD11b to distin*g*uish monocytes (CD45^+^CD11b^+^) and further gated to quantify inflammatory monocytes (Ly6C^hi^) which have a protective role in stroke progression (Garcia-Bonilla et al. 2018). Based on flow cytometry assessment, increased amount of monocytes occurred in ischemic brain postcerebral ischemia when compared to the sham group (Figure 2(a)). Further analyses indicated that the percentage of Ly6C^hi^ monocytes in the MCAO+RIPC group significantly increased in ischemic brain and decreased in peripheral blood in comparison to the MCAO group at 24 h and 72 h after stroke (*P* < 0.01, Figures 2(b) and 2(c)). These results indicated that RIPC accelerated the infiltration of Ly6C^hi^ monocytes into ischemic brain in mice after MCAO.

### 3.3. RIPC Regulated CD86, iNOS, CD206, and Arg-1 Expressions in Mice after MCAO

**Figure 3:**
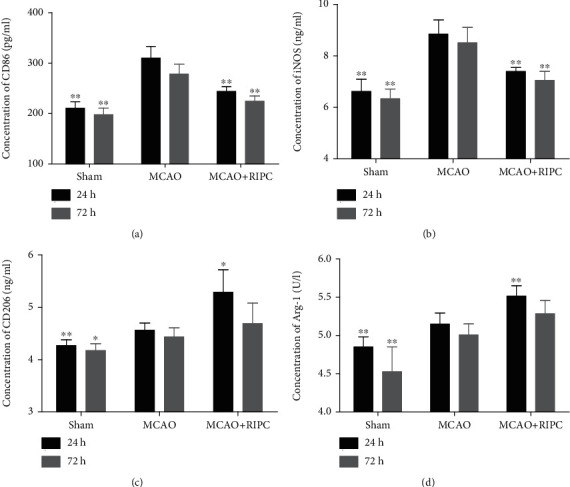
RIPC downregulated the expressions of CD86 and iNOS, while it upregulated the expressions of CD206 and Arg-1 in ischemic brain at 24 h or 72 h after MCAO. The level of cerebral homogenate CD86 (a), iNOS (b), CD206 (c), and Arg-1 (d) at 24 h or 72 h after MCAO (*n* = 6). Values were shown as mean ± SD^∗^*P* < 0.05 and ^∗∗^*P* < 0.01*vs.* the MCAO group.

Overexpression of biomarkers such as CD86 and iNOS is associated with proinflammatory phenotype microglia/macrophage (M1), whereas CD206 and Arg-1 were selected as anti-inflammatory phenotype microglia/macrophage (M2) marker. As shown in Figure 3 ELISA results, the levels of both CD86, iNOS, CD206, and Arg-1 were higher in the MCAO group at 24 h and 72 h in comparison to the sham group (*P* < 0.01). In comparison to the MCAO group, the dramatic enhancement of CD86 and iNOS in brain tissue was inhibited by RIPC (*P* < 0.01, Figures 3(a) and 3(b)). After RIPC, the expression of CD206 and Arg-1 in brain tissue raised significantly at 24 h post-MCAO (Figures 3(c) and 3(d)), whereas the trends were not apparent at 72 h post-MCAO. These results showed that RIPC downregulated the expressions of CD86 and iNOS, while it upregulated the expressions of CD206 and Arg-1.

### 3.4. RIPC Regulated TNF-*α*, iNOS, TGF-*β*, and Arg-1 mRNA Expressions in Mice after MCAO

**Figure 4:**
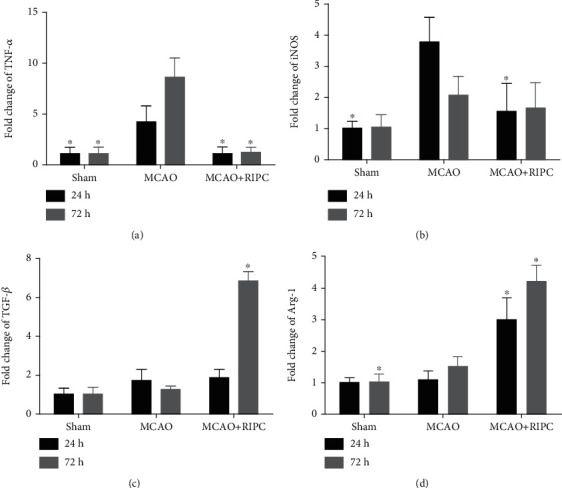
RIPC suppressed the mRNA expressions of TNF-*α* and iNOS and promoted the mRNA expressions of TGF-*β* and Arg-1 in ischemic brain at 24 h and 72 h after MCAO. Real-time PCR of TNF-*α* (a), iNOS (b), TGF-*β* (c), and Arg-1 (d) at 24 h and 72 h after MCAO (*n* = 4). Values were shown as mean ± SD^∗^*P* < 0.05, ^∗∗^*P* < 0.01*vs.* the MCAO group.

To explore the outcome of RPIC on microglia/macrophage polarization, we determined the mRNA expression of TNF-*α*, iNOS (both M1-related factors), TGF-*β*, and Arg-1 (both M2-related factors) by real-time PCR in ischemic brain. As shown in Figure 3, TNF-*α*, iNOS, and Arg-1 increased significantly in the MCAO group compared to the sham group at 24 h or 72 h post-MCAO (*P* < 0.05). In the MCAO+RIPC group, the mRNA expression of TNF-*α* showed a significant decrease at 24 h and 72 h post-MCAO (*P* < 0.05, Figure 4(a)), while the mRNA level of iNOS significantly reduced at 24 h post-MCAO (*P* < 0.05, Figure 4(b)). In contrast, TGF-*β* and Arg-1 were elevated dramatically in the MCAO+RIPC group in comparison to the MCAO group at 72 h post-MCAO (*P* < 0.05, Figures 4(c) and 4(d)). Taken together, these results suggested that RIPC suppressed the mRNA expressions of M1-related factors (TNF-*α* and iNOS) and promoted the mRNA expressions of M2-related factors (TGF-*β* and Arg-1).

### 3.5. RIPC Regulated Microglia/Macrophage Polarization in Mice after MCAO

**Figure 5:**
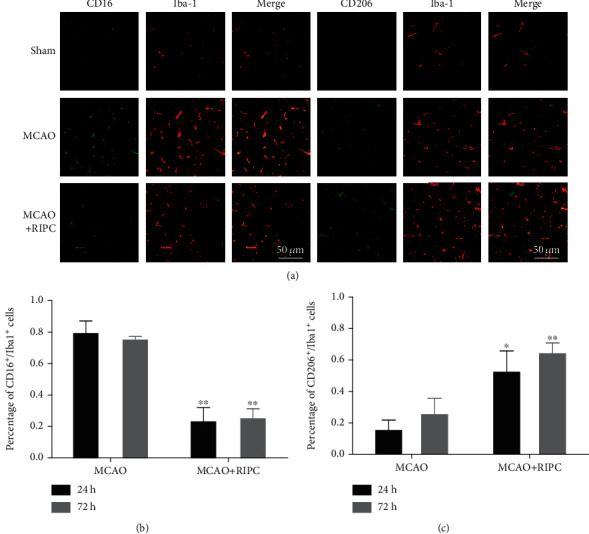
RIPC inhibited proinflammatory microglia and facilitated anti-inflammatory microglia polarization in ischemic brain at 24 h and 72 h after MCAO: (a) representative immunofluorescence staining images of brain sections at 24 h after MCAO; (b) quantification of the percentage of CD16^+^/Iba-1^+^cells at 24 and 72 h after MCAO; (c) quantification of the percentage of CD206^+^/Iba-1^+^cells at 24 and 72 h after MCAO. Data presented as mean ± SD (*n* = 3). ^∗^*P* < 0.05 and ^∗∗^*P* < 0.01*vs.* the MCAO group.

To further evaluate the polarization of microglia/macrophage in ischemic brain, we used Iba-1 to mark resting and activated microglia/macrophage and CD16 and CD206 to mark M1 and M2, respectively [19, 20]. The immunofluorescence staining result showed that levels of the M1 marker CD16 in the MCAO group were higher in Iba1^+^ cells at 24 h and 72 h post-MCAO (Figure 5(b)). When compared to the MCAO group, CD16 and Iba1 coexpression was lower in the MCAO+RIPC group (*P* < 0.01, Figure 5(b)). In contrast, levels of the M2 marker CD206 heightened in the MCAO+RIPC group in comparison to the MCAO group at 24 h and 72 h post-MCAO (Figure 5(c)). Moreover, the MCAO+RIPC group showed more significant difference than the MCAO group at 72 h (*P* < 0.01). These data suggested that RIPC inhibited proinflammatory microglia and facilitated anti-inflammatory microglia polarization in MCAO mice.

### 3.6. RIPC Regulated PPAR*γ* and NF-*κ*B Shifting from the Nuclei to Cytoplasm in Mice after MCAO

**Figure 6:**
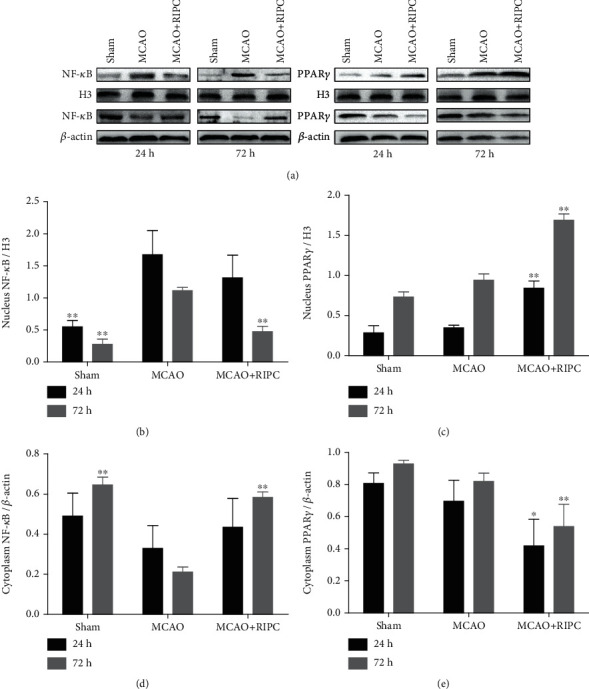
RIPC repressed NF-*κ*B shifting from the cytoplasm to nucleus and stimulated PPAR*γ* shifting from the nucleus to cytoplasm in ischemic brain at 24 h or 72 h after MCAO. Western blot of the nucleus NF-*κ*B (a), cytoplasm NF-*κ*B (b), nucleus PPAR-*γ* (c), and cytoplasm PPAR-*γ* (d) in ischemic brain. Data were shown as mean ± SD (*n* = 3). ^∗^*P* < 0.05 and ^∗∗^*P* < 0.01 *vs.* the MCAO group.

In comparison to the sham group, expression of NF-*κ*B substantially decreased in the cytoplasm but heightened in the nucleus of the MCAO group. After RIPC treatment, cytoplasmic NF-*κ*B increased and nuclear NF-*κ*B reduced (Figures 6(a) and 6(b)). Consistently, the MCAO+RIPC group showed more significant difference than the MCAO group at 72 h (*P* < 0.01).

In the MCAO group, PPAR*γ* levels drastically decreased in the cytoplasm (*P* < 0.01) but enhanced in nucleus in comparison to the sham group. After RIPC therapy, PPAR*γ* reduced in the cytoplasm while increased in the nucleus (Figures 6(c) and 6(d)). The difference between the MCAO+RIPC group and MCAO group was significant at 72 h (*P* < 0.01). Therefore, RIPC inhibited nuclear NF-*κ*B and cytoplasmic PPAR*γ* levels, while it upregulated cytoplasmic NF-*κ*B and nuclear PPAR*γ* expression after MCAO. In other words, RIPC repressed NF-*κ*B shifting from the cytoplasm to the nucleus and stimulated PPAR*γ* shifting from the nucleus to the cytoplasm.

## 4. Discussion

In this present study, our findings indicated that RIPC substantially decreased infarct size, attenuated neurological impairment, promoted Ly6C^hi^ monocyte infiltration into the brain, and suppressed NF-*κ*B-mediated inflammatory response. Furthermore, this neuroprotection was related to promotion of microglia/macrophage M2 polarization through upregulating PPAR*γ* nuclear translocation.

Ren et al. were the first to describe that RIPC conducted on the ipsilateral hind limb reduced the cerebral infarct size after focal cerebral ischemia [21]. An increased number of reports indicate that PIPC is protective from stroke-induced brain injury [22, 23]. Consistent with prior studies, our findings indicated that RIPC not only decreased cerebral infarct size but also promoted neurological role in early stage postischemic stroke.

Monocytes, which are recruited from peripheral blood after ischemic brain injury, can be separated into two categories in mice: inflammatory and patrolling monocytes [24]. The two common types of murine monocytes are classified by their level of Ly6C expression: Ly6C^hi^ and Ly6C^lo^. Chu at el suggested that Ly6C^hi^ monocytes exerted an acute protective influence after the establishment of ischemic stroke and functional deficit that involved promotion of M2 macrophage polarization [16]. Using flow cytometry, we characterized the profile of monocytes circulating in the blood and entering the brain early after stroke. There was a profound increase in the proportion of brain monocytes after stroke. Our data also showed that Ly6C^hi^ monocytes entered the circulation and then the ischemic brain during the acute poststroke period. RIPC improved the percentage of Ly6C^hi^ monocytes in ischemic brain, resulting in beneficial outcome after cerebral ischemia.

Recently, studies have demonstrated that microglia/macrophage can function as double-edged swords in many diseases, depending on their phenotypes [25]. For example, the M2 phenotype ameliorated neuronal survival and tissue repair, while the M1 phenotype hastens neuronal necrosis and aggravates inflammation [26, 27]. And prior analyses have suggested that regulating the polarization of microglia, inhibiting M1 phenotype, and promoting M2 phenotype lead to neuroprotective effects in animal models of ischemic stroke [28, 29]. Similarity, our immunofluorescence data indicated that RIPC increased microglia/macrophage polarization towards M2 phenotype rather than M1 phenotype, as shown by a reduction of CD16^+^/Iba-1^+^cells and increased CD206^+^/Iba-1^+^cells at 24 h and 72 h postreperfusion. Consistent with immunofluorescence results, RIPC promoted the M2-related factor (TGF-*β* and Arg-1) mRNA expression and inhibited M1-related factor (TNF-*α* and iNOS) mRNA expression. ELISA results indicated that RIPC decreased levels of M1 markers (CD86 and iNOS) and increased M2 marker expression (CD206 and Arg-1) at 24 h and 72 h post-MCAO/R.

Overall, these results together suggested that RIPC was protective towards ischemic stroke by encouraging microglia/macrophage polarization to M2 phenotype in MCAO/R mice.

In addition, we investigated the mechanisms of RIPC regulating inflammatory response and microglia/macrophage polarization. The NF-*κ*B signaling pathway has a vital function in stimulating proinflammatory gene expression [30, 31]. Normally, NF-*κ*B is present within the cytoplasm, where it interacts with and binds to inhibitory proteins and stays inactive. In our experiment, cytoplasmic NF-*κ*B was reduced and the protein levels of NF-*κ*B in the nucleus were increased, while RIPC reversed the NF-*κ*B signaling pathway activation, which suggested the anti-inflammatory effects of PIPC. PPAR*γ* is constitutively expressed in microglia/macrophage, acting as a key regulator of microglia/macrophage activation [32]. Activating the PPAR*γ* signaling pathway has a protective function as it reduces neuroinflammation in several diseases (i.e., Alzheimer's disease, Parkinson's disease, and stroke) [12, 33, 34]. There is also evidence showing that activating PPAR*γ* increases the expression of M2 genes (i.e., Arg-1, CD206, and IL-10) in microglia/macrophage [35]. In our study, RIPC after MCAO/R increased PPAR*γ* nuclear translocation, which contributed to the improvement of M2 gene transcription. Thus, we concluded that the neuroprotective effect of RIPC is related to regulation of microglia polarization regulated by the PPAR*γ* pathway. There are many other potential mechanisms of RIPC protecting against ischemic stroke. A study showed that trained immunity regulators are upregulated in liver ischemic pre- and postconditioning [36]. After cerebral ischemia, RIPC might upregulate the canonical and noncanonical inflammasomes to exert protective effect. In addition to the roles of endothelial cells (ECs) in physiological processes, ECs actively participate in both innate and adaptive immune responses [37]. The regulation of RIPC on ECs is also an interesting research direction. Thus, the effects of RIPC on the regulation of trained immunity are worthy of our research in future experiments.

## 5. Conclusion

We demonstrated that RIPC inhibited the inflammatory reaction and enhanced the postischemic M2 polarization of microglia/macrophage after MCAO/R. Furthermore, our results suggested that the underlying mechanisms were mediated through the NF-*κ*B and PPAR*γ* pathway. This finding suggests the pharmacological efficacy of RIPC and provides evidence of clinical application of RIPC in ischemic stroke therapy.

## Data Availability

The data used to support the findings of this study are included within the article.
