# Correction to “Remote Limb Ischemic Postconditioning Protects against Ischemic Stroke via Modulating Microglia/Macrophage Polarization in Mice”

**DOI:** 10.1155/jimr/9753571

**Published:** 2025-11-20

D. Han, J. Wang, L. Wen, M. Sun, H. Liu, and Y. Gao, “Remote Limb Ischemic Postconditioning Protects against Ischemic Stroke via Modulating Microglia/Macrophage Polarization in Mice,” *Journal of Immunology Research* 2021 (2021): 6688053, https://doi.org/10.1155/2021/6688053.

In the article, there is an error in the western blots presented in Figure 6a, introduced during the production process.

Specifically, the ß‐actin bands shown at 72 h on the right‐hand side are duplicates of the PPAR‐γ bands shown at 72 h on the right‐hand side.

The corrected Figure 6 is below:

Figure 6RIPC repressed NF‐κB shifting from the cytoplasm to nucleus and stimulated PPARγ shifting from the nucleus to cytoplasm in ischemic brain at 24 h or 72 h after MCAO. Representative immunoblots of NF‐κB and PPAR‐γ (a). Western blot for expressions of the nucleus NF‐κB (b), cytoplasm NF‐κB (c), nucleus PPAR‐γ (d), and cytoplasm PPAR‐γ (e) in ischemic brain. Data were shown as mean ± SD (*n* = 3).  ^∗^ *P*  < 0.05 and  ^∗∗^ *P*  < 0.01 vs. the MCAO group.

**Figure:**
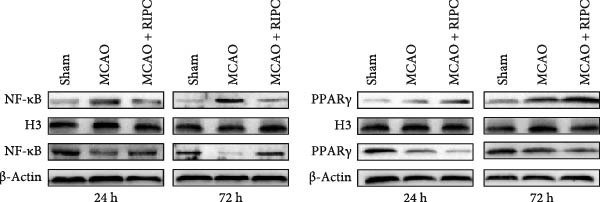
(a)

**Figure:**
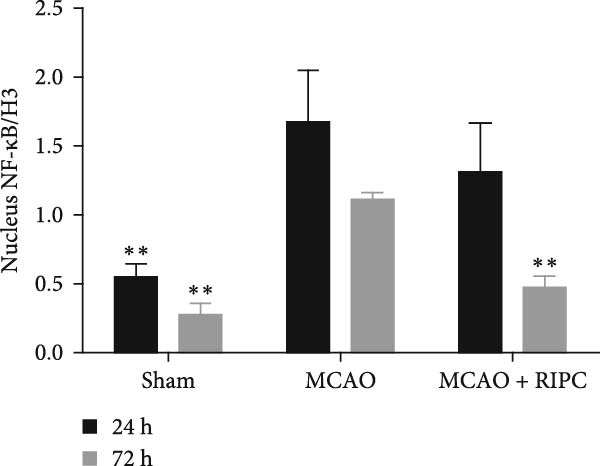
(b)

**Figure:**
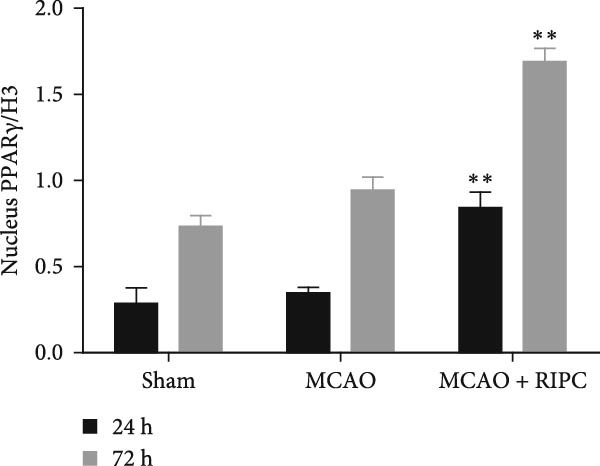
(c)

**Figure:**
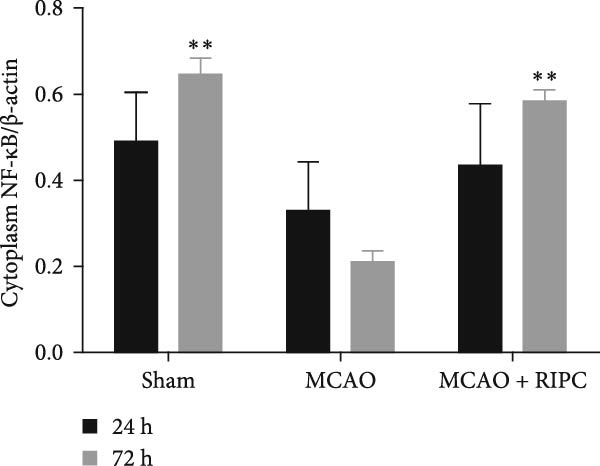
(d)

**Figure:**
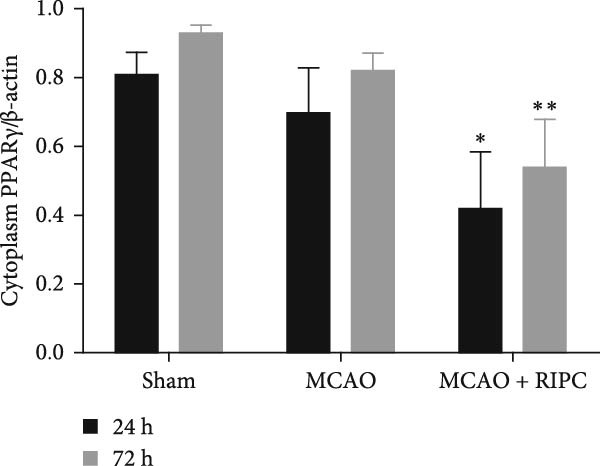
(e)

Additionally, the legend of Figure 6 has been updated for clarity, as shown below:

We apologize for these errors.

